# Knowledge, aptitudes, and preferences in implant dentistry teaching/training among undergraduate dental students at the University of Barcelona

**DOI:** 10.4317/medoral.21741

**Published:** 2017-06-04

**Authors:** Mª Angeles Sánchez-Garcés, Esther Berástegui-Jimeno, Cosme Gay-Escoda

**Affiliations:** 1 MD, DDS, PhD, MS. EBOS, Agregate Professor of Oral Surgery, Professor of Master Degree Program in Oral Surgery and Implantology, Faculty of Dentistry- University of Barcelona, (Spain). Researcher of the IDIBELL Institute, Barcelona, Spain; 2 MD, DDS, PhD. Professor of Dental Pathology and Therapeutics. Director of Master’s Degree Program in Advanced and Experimental Clinical Endodontics”; 3 MD, DDS, MS, PhD, EBOS, OMFS, Chairman and Professor of Oral and Maxillofacial Surgery, Faculty of Dentistry, University of Barcelona. Director of Master’s Degree Program in Oral Surgery and Implantology (EHFRE International University/FUCSO). Coordinator/Researcher of the IDIBELL Institute. Head of Oral and Maxillofacial Surgery Department of the Teknon Medical Center, Barcelona, Spain

## Abstract

**Background:**

Oral implant rehabilitation should be considered a treatment option for any edentulous patient and Implant Dentistry is currently a discipline taught in the undergraduate formation. The level of knowledge acquired and how the students perceive the quality of training in Implant Dentistry could assess to know if it is necessary to improve the syllabus.

**Material and Methods:**

A questionnaire was developed with 11 questions: Basic knowledge (7); Perception of training received (2); Ways in which students would receive training (2). To be responded anonymously and voluntarily for undergraduates students in the Faculty of Dentistry (University of Barcelona, Spain).

**Results:**

One hundred and seven students, 76 third year (Group A) and 31 fourth year (Group B) answered the questionnaire. In Group A, 98.68% of students and in Group B 93.54% believed they were poorly informed; 100% of both groups would prefer to receive more training as part of the degree or as postgraduate training through modular courses imparted by experts (A: 71,05%, B: 70,96%) Training through postgraduate programs or training given by private businesses were the least desirable options (A: 42%, B: 64.51%). Questions about basic knowledge acquired received varying responses, which might indicate a certain level of confusion in this area.

**Conclusions:**

The undergraduate syllabus must be revised to include sufficient content and training to allow the student to indicate implant-based treatments based on evidence. Students would prefer training to be included in the undergraduate syllabus.

** Key words:**Dental implants, dental students, dental education, dental syllabus, implant dentistry.

## Introduction

The use of dental implants for treating complete or partial edentulism is a widely accepted treatment option as a result of its high rate of success (1,2).

Demand for this type of treatment has grown due to the publicity provided by companies supplying dental healthcare services, the growing numbers of independent specialists in implant dentistry, and information spread by friends and relatives, or on the Internet.

Although not all dentists wish or are trained to provide implant-based treatment, it is necessary for all of them to receive sufficient training to inform patients of the advantages and disadvantages (indications, contraindications, prosthetic possibilities in each case, etc.) whenever an implant-based treatment is a viable option within the patient’s individual treatment plan. It is also necessary to adapt the undergraduate syllabus so that the training currently acquired on the degree course in dentistry will be of the same quality as the other disciplines involved in restoring dental function and esthetics and of sufficient scope and quality to allow adequate decision-making treatment that best suited the needs of an individual patient, fulfilling the basic objective of good practice (3).

The degree course syllabus in dentistry at the University of Barcelona, imparted over five years, awards 300 ECTS credits (European Credit Transfer and Accumulation System) that correspond to 25-30 hours per credit, depending on the needs of each individual student as established the European Comission of the European Union foccused on supporting and improving education and training in Europe, one credit is calculated including classroom teaching time, independent study, tutorials, seminars, student-produced work, practice sessions and/or projects, as well as the time needed to prepare and sit exams and tests. Various subjects relating to periodontics, oral surgery or prosthodontics, taken during the third and fourth years of the degree, include transversal teaching on implant dentistry. Students are also given the opportunity to adapt the degree course to their interests by choosing a range of optional subjects awarded 3 ECTS credits each that sums a total of 27 ECTS credits for optional subjects, of which two of the options on offer have some implantology content (“Procedures and clinical techniques applied to implantology” and “Advances in implant and periodontal treatment”) but only one is related to implant dentistry exclusively entitled “Advanced orofacial implantology”, which can be taken during the fourth or fifth year. According to a survey of undergraduate courses in dentistry conducted internationally at different Universities, the total number of teaching hours in implant dentistry varies between 10 and 40 (4).

Implant-based treatments have become usual within dental practice. Universities are responsible for providing students training in theory and in the practical skills necessary to offer patients good quality evidence-based practice (5). The content of the academic year must make it possible to fulfill this objective, and so from the point of view of teaching/training, it is important for university staff to be aware of how students view the quality of the teaching and training they receive.

The aims of this transversal study were: 1. To assess the quality of training given in implant dentistry; 2. To assess how the training given in implant dentistry is perceived by undergraduate dental students; 3. To determine how students believe the syllabus could be improved in relation to this field.

## Material and Methods

- Study population 

The number of students enrolled in the third and four year of the EHEA (European Higher Education Area, Bachelor Degree in Dentistry: equivalent to the DDS in the U.S.A.) is 151 in third and 146 in fourth year. In the last month of lectures, during the 2015-16 academic year, the students were invited to respond to a questionnaire anonymously and voluntarily. This questionnaire was distributed among students who were attending the classroom that day in a lecture without surgical contents (Dental Pathology and Therapeutics), in order to avoid any bias resulting from their involvement in a surgery or prosthodontic subject or by the presence of a teaching staff member related to implant dentistry (E.B.).

- Data collection 

The questionnaire consisted of: 1) Seven items aimed at obtaining information about knowledge, aptitudes, and perceptions related to implant-based treatments in which participants must selected a single answer out of five alternative options ( Table 1, Table 2) Four questions about undergraduates’ perception of the knowledge acquired and the training options that they believed the most adequate in order to improve implant discipline learning ( Table 2). The questionnaire was adapted from an earlier version developed by Chaudhary *et al.* (6) used among a population of dental undergraduates in India removing a question related to economic cost of the implants.

**Table 1:**
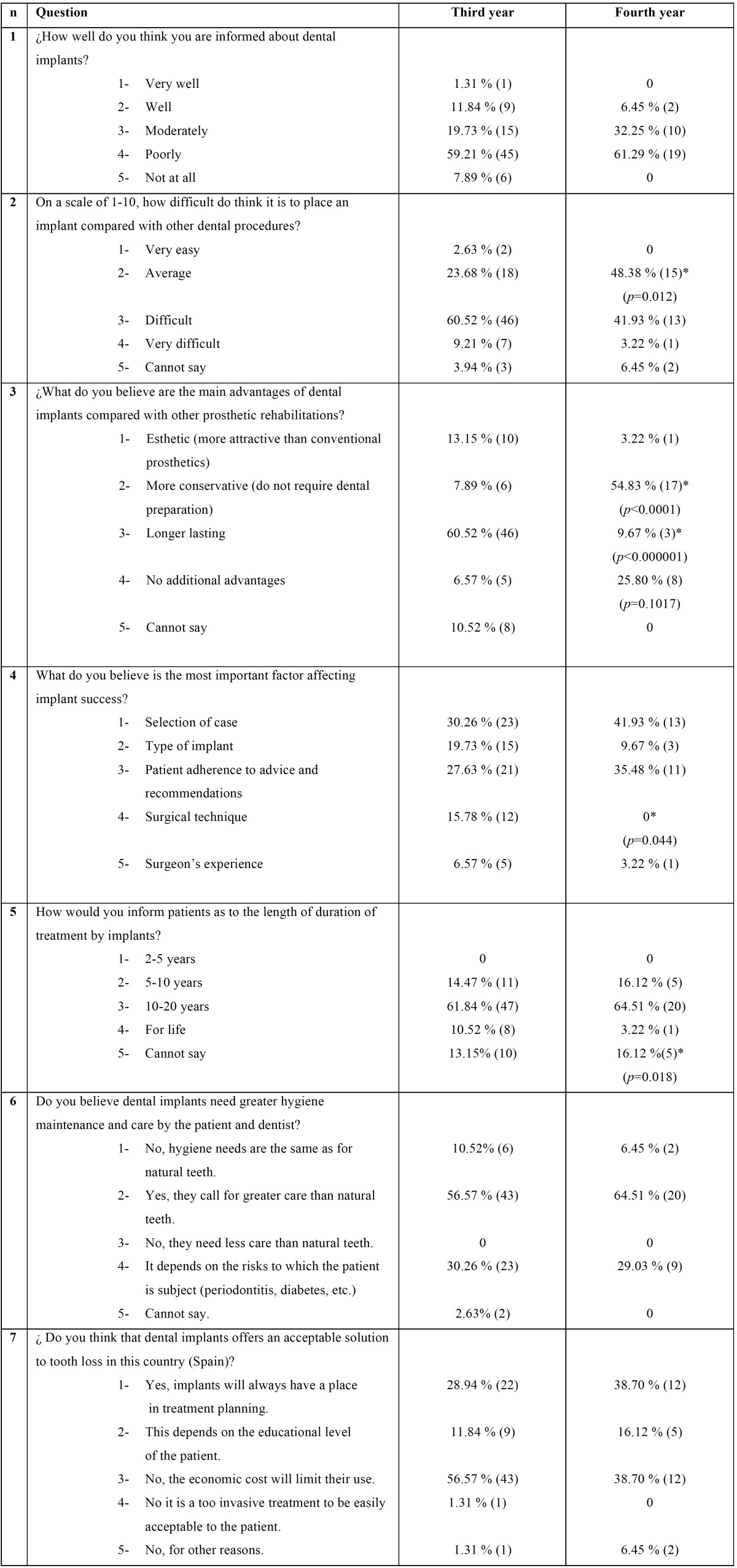
Knowledge, attitudes and perceptions of implant dentistry by undergraduates. Responses annulled because responders selecting more than one option: question 3: 2 cases; question 4: 3 cases. *Statistically significant difference.

**Table 2:**
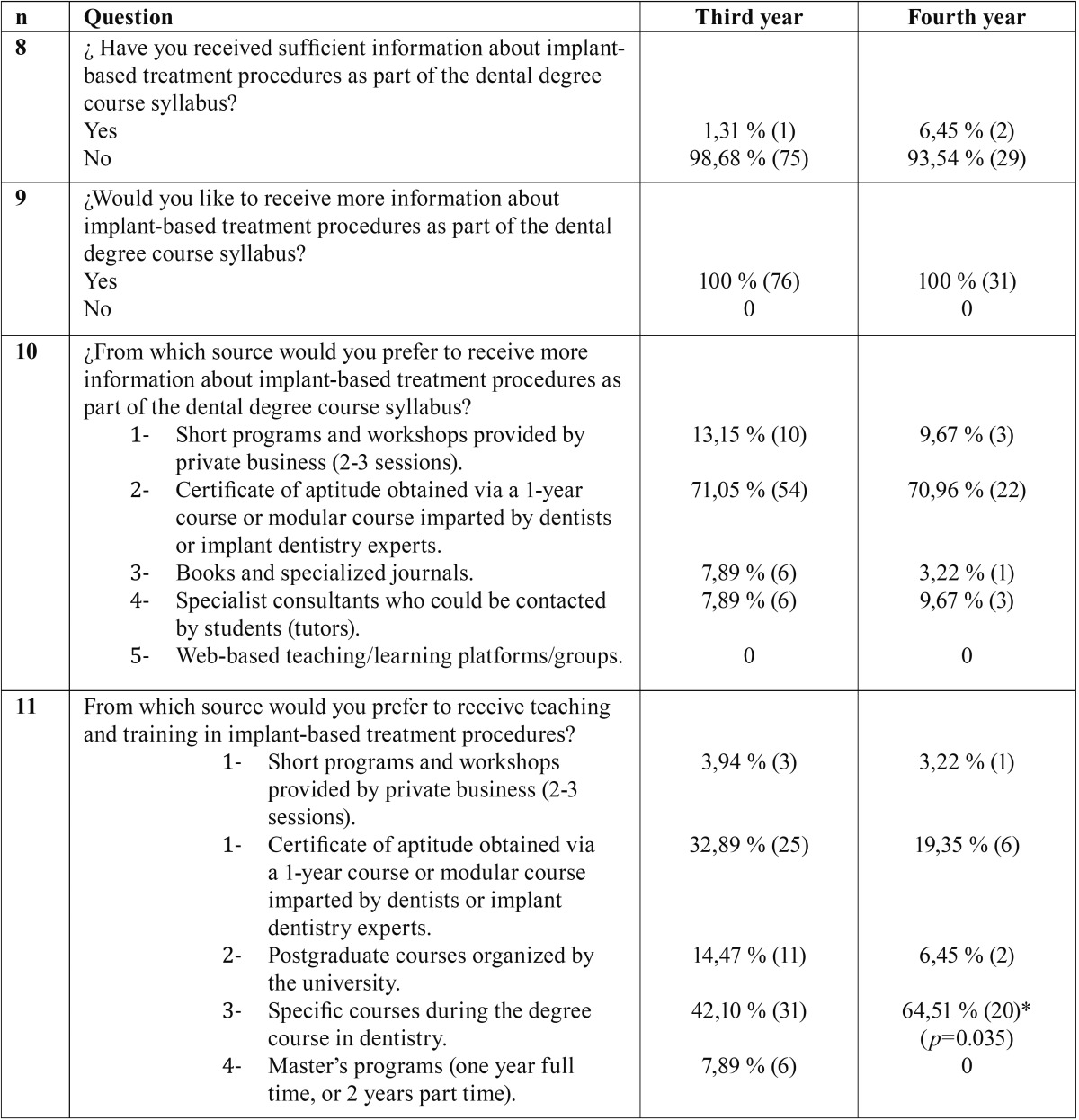
Questions about possible sources of information, teaching or training, and the need for more information about implant-based treatments. Responses annulled because responders gave more than one answer: Question 10: 2 cases, Question 11: 2 cases. *Statistically significant difference.

- Statistical analysis

Data collection and analysis were performed by one examiner (MAS) using the educational statistical software package Stat-Crunch, which is available on line (https://www.statcrunch.com). To evaluate the answers, a descriptive statistical analysis of the qualitative variables and their absolute frequencies and proportions were performed. Differences between proportions were calculated with respect to the answers of each question for each group ( A or B) and between the two study groups, by Chi-square test and Fisher exact test or employing Yate’s correction for continuity, when frequencies were less than five in the answers with only two possibilities (yes or not). Statistical significance was set at *p* < 0.05.

## Results

One hundred and seven questionnaires were analyzed, representing 50,33% of the third course and 21,23% of the fourth curs (36,02%) of undergraduates enrolled in the third and fourth years of the Degree Course in Dentistry). As the questionnaires remained anonymous it was not possible to determine the gender of the participants, although data provided by the Faculty of Dentistry state that the percentage of students enrolled according to gender is 70% males.

-Information about knowledge, aptitudes and perceptions of implant-based treatments.

Respect of the seven questions aimed to determine the students’ level of information about treatments with dental implants ( Table 1), more than half of students in both years considered themselves poorly informed without significant differences between the third and fourth year students (group A: 59.81% and B: 61,29%), and 19.73% and 32,25% ( A and B respectively) believed themselves moderately well informed, with a higher percentage among fourth-year students although without statistically significant difference between the groups. This shows that, in spite of having received more information in the fourth year degree, self-perception of their knowledge did not improve.

With regard to students’ opinion as to the level of difficulty of performing this type of treatment, 48,38% of fourth-year (group B) and 23.68% (group A) of third-year students believed the treatment is of average difficulty, with significant difference between the groups (*p* 0.012). These results were repeated inversely among those students who thought the treatment is difficult to perform, with most third-year students (60.52%) perceiving it as difficult, although the percentage fell to 41.93% as the information received by students increased (fourth-year students).

For questions aimed at determining students’ level of basic knowledge, which would corresponded to basic questions asked by patients when an implant-based treatment is proposed, more than an half of fourth-year students (54.83%) believed that implant treatments are less invasive than dental preparation, while only 7.89% of third-year students thought this (*p*<0.0001), although they saw implants as a longer-surviving treatment than fourth-year undergraduates (*p*<0.00001). But when asked to state the life expectancy of implants, both groups chose the same option (10-20 years). A difference in perception was shown by the question as to whether implant-based treatments are more advantageous than treatments based on natural teeth, in which fourth-year students believed that implants did not offer more advantages with significant difference (*p* =0.017) respect to those of the third-year. Both groups agreed that implant-based rehabilitations required more maintenance than restorations supported by natural teeth.

In response to the question as to the most important factor determining implant success, both groups answered in similar proportions, without significant differences except for surgical technique (*p*=0.044). Students believed that success is related to all the options although case selection, and fulfillment of recommendations made to the patient were awarded more importance ( Table 1). Three answers were eliminated because fourth-year students group selected more than one option.

Respect to whether students believed that implants are an acceptable solution to edentulism in our country (Spain), fourth-year students thought that implants would always play a part in treatment planning, and the factor that would limit their use was their economic cost; third-year students also believed mainly that the use of implants would be limited for reasons of cost.

-Perception of knowledge acquired and the training options available best suited to improving knowledge/training.

Almost one hundred per cent of third-year students and 93.54% of fourth-year students believed that the information received during the degree course in dentistry was insufficient. Both groups agreed that they would have liked to receive more information during undergraduate training (100%) ( Table 2).

With regard to the best way of acquiring knowledge/training, both groups preferred courses of one-year duration or modular courses imparted by experts in the field of implant dentistry (71% for both groups).

Fourth-year students (group B) saw a specific one-year course in implant dentistry included as part of the degree syllabus as the best way to acquire learning in this field (64,51%). But third-year students did not believe that (42,10%), with statistically signifi-cant difference between the groups (*p*=0.035).

The least desirable learning/training options for both groups were enrollment in a university post-graduate course (one year full time, or 2 years part time) or training provided by private brands.

## Discussion

According to a study made by the Millennium Research Group (Toronto, Canada), among European countries, Spain places the second highest number of implants per year – approximately 130.000 (7). As the Spanish Society of Implantology (SEI) published, 500,000 implants were inserted in 2015 (8), and in 2016 it is estimated that the implant dentistry sector will be 31.7% of sales in the dental supplies market, which represents an overall market value of 642 million euros, according to sources in the Spanish Federation of Healthcare Technology Companies (FENIN) (9). This massive growth in demand for dental implants is mainly due to greater public awareness of this therapeutic option, mainly as a result of information passed by friends and relatives and above information provided by dentists, according to SEI published (8).

The “White Paper on Implant Dentistry in Spain,” a survey conducted among a range of scientific societies related to implant dentistry in Spain, over 90% of the professionals consulted believed that implantology teaching in undergraduate syllabuses at Spanish universities is of poor or bad quality (8), an opinion shared by some 60% of the students consulted in the present study. Nevertheless, 51.5% of dentists believe that the quality of post-graduate implantology teaching is good or excellent and 66.7 % believe that training given by private brands is good or very good (8).

According to the results of the present transversal study, the fourth-year students who responded to the questionnaire, implantology training within the undergraduate program is the preferred option to learn this specific subject area, while third-year students preferred teaching as a specialized one-year post-graduate course imparted by experts, out of the university regardless of whether this would be structured as modules or as continuous training. The least desirable option was thought to be training within a full-time or part-time postgraduate program of one or two years (corresponding to Diplomate or Master degree program) (6.45%) or training provided by private brands (3.22%), on the contrary to the opinion of practicing professionals. It is possible that only students who had decided to focus his future on this specialization chose this option.

The 1st European Consensus Workshop on Implant Dentistry University Education agreed that an implant dentistry postgraduate program structured as twelve weeks full time could lead to an Intermediate Certificate that would provide the professional clinical skills corresponding to level A of SAC (Straightforward, Advanced, Complex) Classification in Implant Dentistry (10) or alternatively as a two-year academic program of part-time flexible study. With regard to the content of undergraduate study in implant dentistry, another working group at the 1st European Consensus Workshop on Implant Dentistry University Education, proposed that teaching content should match that of the University of Zurich (Switzerland), which consists of: osseointegration, materials, clinical and radiological diagnosis, treatment planning, implant/soft tissue relationships, esthetic considerations, types of prostheses, surgical procedures, surgical complications, soft tissue and bone management (bone regeneration), biomechanical aspects of rehabilitation, etiology, pathogenesis and prevention of peri-implantitis and implant maintenance. This content would allow the student to appreciate the degree of difficulty of each treatment and facilitate the provision of adequate information to the individual patient (5). This is all essential teaching/learning content in contemporary contexts and should be adapted for inclusion in the undergraduate syllabus and so meet the needs expressed by undergraduate students.

Another interesting datum that highlights the need to train students in the field of implant dentistry, according to the SEI White Paper, it would appear that in the future implant treatments will mainly be performed in general dental clinics (87.4 %) rather than in specialized implantology clinics (7.3%) (8). It seems that most implants are placed in dental clinics that offer integrated dental healthcare, where the ability to assess the indications for implant-based treatment with adequate criteria and to inform the patient accurately respect to the risks and benefits of this type of treatment must be guaranteed. In this context, the recently graduated general dentist must be prepared to perform these basic duties as soon as they enter into professional practice. Nevertheless, according to a survey conducted by the SEI, 57% of patients feel that they were well informed about the real risks and benefits of implant-based treatments (8).

Data obtained by the present questionnaire, states there was a wide disparity of opinion about general concepts in implant treatments such as: the estimated duration of implant treatment, follow-up requirements, or the most important factor governing the success of implant-based treatments. As the degree course advanced and students progress in their learning, they agreed that implant treatments are less aggressive than dental preparation procedures; this datum (59.8%) coincides with a population of dental undergraduates in India (6). Interestingly, this is the main reason why patients request this type of treatment, Ken (11) report that 32% of patients declared this to be the reason for choosing implant-based treatment, being the second reason a rejection of the option of removable prostheses, and the third because the treatment had been recommended by someone they knew. In a similar vein, the study also showed that most patients ignore some very important factors such as the fact that smoking has a negative impact on the life expectancy of the implant (48%), the risk of peri-implantitis (80%), or the fact that they believe that an implant-supported prosthesis has a long life of 20-25 years (52%) (11). Over 60% of the undergraduates who responded to the present questionnaire believed the life of these treatments is between 10 and 20 years, without significant difference between the groups, in contrast to the study by Chaudhary *et al.* (6) in which only 39.8% answered the question in this way. Such opinions confirm the need for factual information so that undergraduates and post-graduates may give the patient realistic expectations for treatment, factors involved in the treatment, and about the risks involved in each individual case (11).

Regarding the key factors governing implant success, more fourth-year students chose case selection as the key factor (41.93%) while other studies have obtained 65.1% (6). For the second most important factor, 35.48% of our student sample believed that treatment success was highly dependent on patients following advice and recommendations given by the dentist. Other possibilities such as implant type, or surgical technique, appeared less important to the students, but perhaps choosing a single answer was difficult, particularly when they was better informed; this would explain why some fourth-year students gave more than one answer.

Another questionnaire done by Aljohani and AlGhamdi (12) with 21 questions was distributed among recent graduates set out to obtain information about the levels of basic knowledge among this population. The study reached the same conclusions in most aspects, confirming that there is a lack of understanding and some confusion such are indications and risk factors. Indeed, 78.8% of the graduates believed that they had not received sufficient teaching/training in implant dentistry and 100% believed that their knowledge was insufficient. However, Chaudhary *et al.* (6) reported that 75% of the students surveyed believed that they were well or moderately well informed.

The teaching/training in dental implantology on the degree course in dentistry at the University of Barcelona (Spain) imparted over 5 years, consists of one hour of theory teaching, and one hour of “hands on” practical training, as part of the subject “oral surgery” during the third-year and although implant dentistry is referred to in other subjects during the third year, but the topic is not included in their specific program content. The subject “Clinical oral surgery and implant dentistry”, consisting of 14 hours (7 lessons of two hours duration), is given in the fourth year; two further “topic blocks” are included in the subjects “Clinical prosthodontics and craniomandibular dysfunction” and “Clinical periodontics,” although the number of teaching hours on implantology is not specified in the published syllabus.

As for the optional subjects on offer, the student may take 15 hours theory learning/teaching and 9 hours practical training (“Advanced orofacial implantology”), as well as 1 hour of prosthodontic implantology treatment program, and a further hour within another subject dealing with maxillofacial surgery. The topics detailed in the syllabus range from basic principles, diagnosis, indications, surgical techniques, types of implant-supported prosthesis, immediate implants, bone regeneration techniques and biomaterials, and complications, which fulfill recommendations made by the 1st European Consensus Workshop on Implant Dentistry University Education (10).

It is clear from the results of the present study that third-year training in implant dentistry is insufficient, which is unsurprising as most of this content is given in the second half of the fourth year or as an option during the fifth year. This explains the major differences in perceptions of training/teaching between the groups who responded to the questionnaire. Nevertheless, almost all students believed that they had not received sufficient teaching/training (98,68% third-year; 93.54% fourth-year) and all believed that this content should be imparted during the university degree course either as a specific subject or transversally. Most universities that offer implant dentistry studies do so during the fourth (15%), fifth (39%), or sixth (36%) year of the degree (4).

In a survey conducted at 92 Faculties of Dentistry, 86% offered pre-doctoral studies in implant dentistry in U.S.A., Europe, Asia, South America, and Africa, and the remaining 14% defined the reasons why such courses were not initiated as: insufficient time to develop the subject within the syllabus, lack of budget, importance allotted to this area as a part of post-graduate teaching/training, or lack of qualified teaching staff (4).

With regard to the best ways of imparting subjects content in this field, it is useful to apply Miller’s pyramid as model for the teach/learning process, whereby the levels of knowledge and training increase and develop gradually as the degree course advances (13). In an analysis of how to impart/acquire knowledge in implant dentistry teaching/learning and how to evaluate students, Mattheos *et al.* concluded that the most adequate approach is to stimulate interest in self-learning and to foster self-recognition of the gaps in knowledge that the individual may suffer, so that they may direct his interest into correcting any deficiency through a positive attitude to lifelong learning (14). This does not mean that the teaching/training syllabus for implant dentistry should not be discussed and agreed on, at least among universities in the European Union, which would allow student and teaching staff greater mobility under equalized conditions.

## Conclusions

- The undergraduate syllabus in dentistry must be revised to include sufficient knowledge and practical experience to allow the student to indicate implant-treatments based on evidence, and to inform the patient about the real risks and possible solutions relevant to his individual case.

- Students believe that they are not well informed about implant-based treatments and would prefer teaching/training in this field to be given as part of the degree syllabus.
